# Aptamer-Based Therapeutics: New Approaches to Combat Human Viral Diseases

**DOI:** 10.3390/ph6121507

**Published:** 2013-11-25

**Authors:** Ka-To Shum, Jiehua Zhou, John J. Rossi

**Affiliations:** 1 Department of Molecular and Cellular Biology, Beckman Research Institute of City of Hope, 1500 East Duarte Rd, Duarte, CA 91010, USA; E-Mail: jzhou@coh.org; 2 Irell and Manella Graduate School of Biological Sciences, Beckman Research Institute of City of Hope, 1500 East Duarte Rd, Duarte, CA 91010, USA

**Keywords:** SELEX, aptamers, virus, HIV-1, RNA nanotechnology

## Abstract

Viruses replicate inside the cells of an organism and continuously evolve to contend with an ever-changing environment. Many life-threatening diseases, such as AIDS, SARS, hepatitis and some cancers, are caused by viruses. Because viruses have small genome sizes and high mutability, there is currently a lack of and an urgent need for effective treatment for many viral pathogens. One approach that has recently received much attention is aptamer-based therapeutics. Aptamer technology has high target specificity and versatility, *i.e.*, any viral proteins could potentially be targeted. Consequently, new aptamer-based therapeutics have the potential to lead a revolution in the development of anti-infective drugs. Additionally, aptamers can potentially bind any targets and any pathogen that is theoretically amenable to rapid targeting, making aptamers invaluable tools for treating a wide range of diseases. This review will provide a broad, comprehensive overview of viral therapies that use aptamers. The aptamer selection process will be described, followed by an explanation of the potential for treating virus infection by aptamers. Recent progress and prospective use of aptamers against a large variety of human viruses, such as HIV-1, HCV, HBV, SCoV, Rabies virus, HPV, HSV and influenza virus, with particular focus on clinical development of aptamers will also be described. Finally, we will discuss the challenges of advancing antiviral aptamer therapeutics and prospects for future success.

## 1. Introduction

Vaccination is the most effective means to prevent individuals from being infected with pathogenic viruses [1]. However, some viruses, such as HIV-1 and hepatitis C virus, can evade the immune system, and thus impede the effectiveness of vaccines for those viruses [2,3,4]. Therefore, antiviral small molecule inhibitors that inhibit critical steps in the virus lifecycle in infected individuals are critically needed in the battle against virus infections. These inhibitors could curb the virus number in the body by interfering with viral entry into host cells, the function and assembly of viral replication machinery or the release of viruses to infect other cells [5]. Ideally, these antiviral agents should completely eradicate viruses from the body without affecting normal cellular metabolism. However, these features have not yet been achieved because of two main problems associated with use of these drugs: (1) the emergence of resistant viral strains and (2) cytotoxicity to host cells [6,7,8]. Some viruses, such as HIV-1, replicate its genome with high error rate [9]. These mutations in the viral genes that code for surface antigens and enzymes in the replication components often confer drug resistance capabilities to viruses [10]. Also, cytotoxicity often arises because antiviral drugs are usually designed to target and inhibit certain functional motifs of a viral protein. These motifs share a high degree of amino acid sequence similarity across different species and associated with conserved functions. One example demonstrating the motif similarities between viral and human proteins is helicases in which their DEAD-box domain is largely conserved in HCV helicase and human DDX3 RNA helicase [11]. Although off-target cross reactivity often leads to mild side effects, sometimes they are serious and can have a major effect on health. For example, the HIV-1 reverse transcriptase inhibitor 3'-azido-3'-deoxythymidine (zidovudine) is a nucleoside analogue that competes with natural deoxynucleotides (dNTPs) and is incorporated into the growing DNA chain by viral reverse transcriptases. Treatment with zidovudine delays the progression of AIDS, but does not clear the virus because drug resistant mutants usually arise [12,13]. Moreover, long-term, high-dose treatment with zidovudine can cause serious complications, such as anemia, neutropenia, hepatotoxicity, cardiomyopathy and myopathy [12,14,15,16].

Aptamers are *in vitro* evolved nucleic acids that are capable of performing a specific function [17,18]. The process to identify a functional ligand from a vast population of random sequences is called Systematic Evolution of Ligands by Exponential enrichment (SELEX). Typically, an initial combinatorial library contains a central random region with 30 to 70 nucleotides flanked by a fixed sequence at both ends. The fixed sequence is used for PCR amplification during each SELEX round. Random sequences with at least 10^12^ entities represent extraordinary molecular diversity and structural complexity to screen high affinity and bioactive aptamers to the target. To date, a dozen of SELEX methodologies have been developed in isolating aptamers against purified proteins or even whole cells (or whole viruses) [19,20,21]. The use of purified proteins as selection targets has the advantage of easy control to achieve optimal sequence enrichment during the SELEX. But whole cell or virus selection is preferred, when the biomarker is unknown. Moreover, since the target protein may be present in a modified form or exist as a protein complex that may be masked and therefore inaccessible to the aptamers, it reflects a more physiological condition when the protein is displayed on the cell surface rather than isolated as purified proteins.

Generally, SELEX comprises of cycles of four sequential steps: (1) binding to the target; (2) partition of target-bound aptamers; (3) recovery of target-bound aptamers; and (4) amplification of recovered sequences [22,23,24]. The selection cycle is complete when a functional aptamer sequence is enriched among the random sequence library. Since the inception of SELEX technology two decades ago, the extraordinary diversity of molecules screened in this manner has led to the discovery of aptamers that bind with exquisite specificity and extraordinary strength [25,26]. Macugen (Pfizer), which is used to treat age-related macular degeneration, was the first aptamer therapeutic approved by United States Food and Drug Administration (FDA) and has proven to be a milestone in the aptamer history [27,28]. Many novel aptamers are currently being evaluated in clinical trials for treating life-threatening diseases, such as acute myeloid leukemia, renal cell carcinoma, acute coronary syndrome, and choroidal neovascularization [29,30,31,32,33]. In addition, because aptamers can easily be conjugated to chemicals and manufactured, the use of aptamer chimeras for targeted delivery and enhanced potency of secondary agents has progressed rapidly [23,34,35,36,37,38]. In this review, we will focus on the recent progress and prospective use of aptamers against a variety of human viral pathogens; representative examples of aptamer chimeras will be highlighted. Finally, we will discuss the challenges of advancing antiviral aptamer therapeutics and the prospects for future success.

## 2. Aptamers as Antiviral Therapeutics

Aptamers are single-stranded non-coding nucleic acids screened by SELEX to perform a defined function by forming a complex structure complementary to their targets. Because of their versatility in structure and function, aptamers have many advantages over small molecules and biologics for therapeutic applications, particularly as antiviral therapeutics [26,39]. First, traditional antiviral small molecules, such as HCV protease inhibitors (boceprevir, Victrelis^®^, Merck, Whitehouse station, NJ, USA) and HIV integrase inhibitor (elvitegravir, GS-9137, Gilead, Foster City, CA, USA), fit into crevices on protein surfaces, especially into the active site of enzymes to inhibit their catalytic activity, while aptamers can also form clefts that bind protruding parts of protein [26,40]. Thus, aptamers can bind more specifically and tightly because they extend surface contact with their targets, and thereby disrupt protein-protein or protein-nucleic acid interactions more effectively than small molecules [41]. For example, one HIV reverse transcriptase (RT) aptamer is able to mask ~2600 Å^2^ of the enzyme’s surface, which is likely to slow down the evolution of resistant viruses [41,42,43].

Second, because the folding of nucleic acid aptamers is mainly governed by Watson and Crick base-pairing, aptamers can intrinsically form various loops and diverse thermodynamically stable structures in a highly programmable way [44]. Thus, aptamers can serve as building blocks for bottom-up fabrication of an aptamer chimera system [45,46]. For example, aptamers and other nucleic acid therapeutics can link with a nanovector that has polyvalent functionalities [46,47,48,49]. Several novel aptamer-based chimeras, such as aptamer-aptamer chimeras, aptamer-ribozyme chimeras and aptamer-siRNA chimeras, have been designed [50,51,52]. An innovative use of such chimeras is as drug delivery carriers for cell- or tissue-specific targeted delivery [34]. These cell-internalizing aptamers act as “smart bombs” that only target a particular cell population and deliver their therapeutic cargos specifically into the cell, thereby offering enhanced therapeutic efficacy and reduced cellular toxicity [37].

A third advantage is that potent aptamers can be identified through the iterative SELEX process and evolved in a test tube within a month. Canonical SELEX requires a purified soluble form of the target proteins; however, if the purified protein does not fold into a stable conformation or the native protein exists as a protein complex that may be inaccessible for aptamer screening, then a cell-SELEX strategy that uses whole living cells or inactivated viruses as targets may be useful for aptamer identification [36,53,54,55]. Many aptamers were developed against viral proteins (e.g., HIV reverse transcriptase and HCV NS3), nucleic acid elements (e.g., HIV TAR, HCV IRES), as well as the entire viruses (e.g., influenza A virus) (Table 1). In addition, since the cell-SELEX approach relies on the differences between two populations of cells (infected cells *versus* healthy cells), it can be performed when the target is unknown and without going through the tedious process of protein expression and purification [53]. Moreover, large-scale manufacture of high quality current good manufacturing practice (cGMP)-grade nucleic acids is possible through solid-phase chemical synthesis, and aptamers, composed entirely of nucleic acids, are regarded as chemical drugs by FDA instead of biologics. These features all speed up the process of aptamer research and development.

In summary, aptamer technology is well-suited for treating viral infections, and there are now many examples, illustrating that a wide range of viruses can be inhibited by aptamers and have potential for clinical applications.

### 2.1. Inhibition of Human Immunodeficiency Virus-1

**Figure 1:**
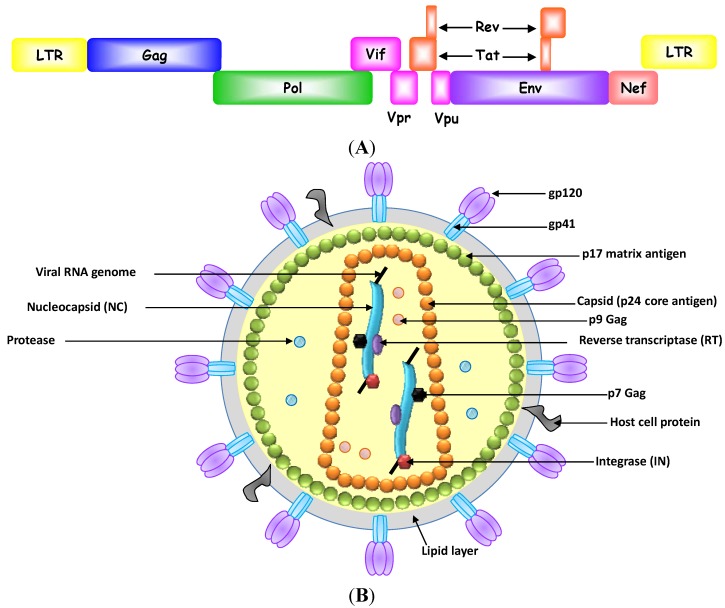
(**A**) HIV-1 genome and (**B**) HIV-1 virion and potential antiviral targets.

HIV-1/AIDS is a chronic disease of the human immune system. Although combination antiretroviral therapy (cART) can slow the progress of the disease and reduce the risk of death and disease complication, it is not curative. Moreover, many patients do not tolerate cART because of its severe side effects, and it is unaffordable for patients in developing countries [56]. In this regard, aptamers have been considered an alternative or adjuvant to the chemical antiviral agents in cART to overcome these limitations [30,57,58]. To date, highly specific, nucleic acid-based aptamers that target various parts of HIV-1 genomes and HIV-1 proteins, including HIV-1 reverse transcriptase (RT), integrase, nucleocapsid, Gag, gp120, TAR, Rev and Tat, have been isolated and shown to effectively suppress viral replication (Figure 1) [57,59]. These aptamers are discussed further below.

#### 2.1.1. HIV-1 Reverse Transcriptase

HIV-1 RT is a key, multifunctional enzyme during the retroviral life cycle. It has a DNA polymerase activity on both RNA and DNA templates and a ribonuclease H activity on RNA-DNA hybrid template [60]. HIV-1 RT catalyzes conversion of the single stranded genomic viral RNA into double stranded proviral DNA, which in turn is integrated into the host genome. 

Currently, RT is one of the main drug targets for HIV/AIDS, and many nucleoside inhibitors (e.g., AZT, 3TC, ddI, ddC, d4T) and non-nucleoside inhibitors (e.g., snevirapine, delavirdine, efavirenz) are currently used in patients [61,62].

**Table 1:** List of representative antiviral aptamers and their properties.

Virus	Aptamer Selection Target	Aptamers	Binding Affinity, K_D_	Inhibitory capabilities	References
HIV	Reverse transcriptase	Pseudoknot; RNA	25 pM	Inhibition of polymerase activity; 95% reduction of HIV particle formation by transient expression of aptamers	[42,63,64]
HIV	Drug resistant Reverse transcriptase mutant 3	M302; N-methyl-isatoicanhydride modified RNA	30 nM	No inhibition of polymerase and RNase H activities	[65]
HIV	Reverse transcriptase	ODN112; G-quadruplex DNA	N.A.	Inhibition of RNase H and polymerase activities with IC_50_ = 500 nM; Reduction of viral infectivity in cell culture with IC_50_ < 100 nM	[66]
HIV	RNase H domain of Reverse transcriptase	R12-2; Thiolated DNA aptamer	70 nM	Inhibition of RNase H activity; Reduction of viral infectivity in cell culture with IC_50_ < 100 nM	[67]
HIV	Reverse transcriptase	37NT; DNA	660 pM	Inhibition of primer-template binding and polymerase activity	[68]
HIV	Reverse transcriptase	RT1t49; DNA	~1 nM	Inhibition of polymerase activity with IC_50_ = 0.3 nM	[69,70]
HIV	Reverse transcriptase	PF1; DNA	82 nM	Inhibition of polymerase activity with IC_50_ = 60 nM	[71]
HIV	Reverse transcriptase	RU25-80;	N.A.	Inhibition of primer extension with IC_50_ = 60 nM and viral replication. Dependent on the nonpseudoknot UCAA motif	[72]
		RNA			
HIV	Reverse transcriptase	6/5 asymmetric loop;	N.A.	Inhibition of primer extension *in vitro*.	[73]
		RNA		Inhibition of viral replication in cell culture	
HIV	Integrase	93del; G-quadruplex DNA	N.A.	Inhibition of viral entry, reverse transcriptase and integration activities; Inhibition of cell fusion in cell at 1 µM	[74,75]
HIV	Integrase	T30695; G-quadruplex DNA	N.A.	Inhibition of integrase activities with IC_50_ < 100 nM	[76]
HIV	DP6 truncated Gag lacking p6 and the	DP6-22; RNA	100 nM	Inhibition of Gag-genomic interactions that negatively affects RNA transcription, processing or stability	[77]
	*N*-terminal myristate				
HIV	Nucleocapsid	N70-13; RNA	0.6 nM	Inhibition of nucleocapsid and HIV psi-RNA interaction	[78]
HIV	Nucleocapsid	N50-20; RNA	0.5 nM	Not Determined	[79]
HIV	Gp120 of HIV strain HXB2	J58; 2' Fluoro modified RNA	210 nM	No neutralization of infection	[80]
HIV	Gp120 of HIV-1_BAL_	B40; 2' Fluoro modified RNA	21 nM	Inhibition of gp120-CCR5 interactions	
HIV	Gp120 of HIV-1_BAL_	UCLA1 (truncated B40); modified RNA with an inverted thymine at 3' end and a dimethoxyltrityloxy-(CH_2_)_6_-SS-(CH_2_)_6_-phospholinker at 5' end	150 pM	Neutralization of isolated of R5 strains with IC_50_ < 100 nM;	[81]
				Synergistic effect with a gp41 fusion inhibitor	
				and an anti-CD4 antibody	
HIV	Gp120 of HIV-1BAL	A-1; 2' Fluoro modified RNA	52 nM	Conjugation with siRNAs targeting HIV *tat/rev* and TNPO3 to inhibit HIV-1 infection in primary human peripheral blood mononuclear cells and humanized mice	[82,83]
HIV	Tat-1- derived peptide	RNA Tat; RNA	120 pM	Competition with TAR for sequestering Tat-1 in cell culture	[84]
HIV	Rev	RBE(apt); RNA	N.A.	Conjugation with ribozyme targeting HIV *Env* for gene therapy	[85]
HIV	TAR	IV04; 2'-O-Methyl modified DNA	20 nM	Disruption of TAR secondary structure by formation of RNA-DNA kissing complexes	[86]
HIV	TAR	R-06_24_; RNA	32 nM	Formation of TAR RNA-aptamer complexes by 8-nt complementary base-pairing	[87]
HIV	TAR	a1.16; RNA	17 nM	Formation of TAR RNA-aptamer complexes by 5-nt complementary base-pairing	[88]
HIV	TAR	B22; DNA	50 nM	No inhibition of TAR function	[89]
HCV	NS3	G6-16; RNA	238 nM	Inhibition of protease activity with IC_50_ = 3 µM	[90]
HCV	Truncated protease domain of NS3	G9-I; RNA	10 nM	Inhibition of protease activity with IC_50_ = 100 nM	[91]
HCV	Helicase domain of NS3	G5; RNA	25 nM	Inhibition of helicase activity with IC_50_ = 50 nM	[92]
HCV	NS5B∆C55	B.2; RNA	1.5 nM	Inhibition of HCV RNA polymerase in a non-competitive manner; IC_50_ = 10 nM	[93]
HCV	NS5B	27v; DNA	132 nM	Competition with the RNA template for binding to the RNA polymerase and blocked both the initiation and the elongation of RNA synthesis; IC_50_ = 190 nM	[94]
HCV	NS5B	R-F t1; 2' Fluoro modified RNA	8 nM	Competition with the RNA template for binding to the RNA polymerase and blocked both the initiation and the elongation of RNA synthesis	[95]
HCV	NS5B	P-58; RNA	570 nM	Interference with HCV replication by targeting the essential 5BSL3.2 domain within the *cis*-acting replication element; IC_50_ =185 nM	[96]
HCV	Domain II of IRES	2-02; RNA	11 nM	Inhibition of IRES-dependent translation	[97]
HCV	Domain III-IV of IRES	3-07; RNA	9 nM	Inhibition of IRES-dependent translation	[98]
HCV	Entire IRES	AP50; RNA	5 nM	Inhibition of IRES-dependent translation	[99,100]
HBV	Surface antigen	HBs-A22;RNA	N.A.	Inhibition of receptor binding	[101]
HBV	Truncated polymerase protein	S9; RNA	N.A.	Competition with RNA to inhibit P protein binding to	[102]
				ε signal on pgRNA	
SCoV	Helicase	NG8; Modified DNA	5 nM	Inhibition of nucleic acid unwinding activity; IC50 = 91.0 nM	[103]
SCoV	Helicase	ES15; RNA	N.A.	Inhibition of nucleic acid unwinding activity; IC50 = 1.2 nM	[104]
Influenza A virus	Hemagglutinin [HA-(91-261) from H3N2]	A22; DNA	N.A.	Inhibition of receptor binding; 95% reduction of viruses in mice	[105]
Influenza A virus	Hemagglutinin	A10; DNA	Strong binding	Inhibition of receptor binding, dose-dependent inhibition demonstrated	[106]
	(HA from H5N1)		measured by ELISA		
Influenza A virus	Whole H3N2 virus	P30-10-16;	0.2 nM	Inhibition of receptor binding; 95% inhibition of viral fusion efficiencies in the presence of 5 µM aptamers	[107,108]
	[A/Panama(H3N2) strain]	RNA			
Influenza A virus	Hemagglutinin	HA68; DNA	7 nM	Inhibition of receptor binding; complete inhibition of the agglutination of RBC in the presence of 2.5 µM aptamers	[109]
	(HA from H3N2)				
Influenza A virus	Hemagglutinin	C7; DNA	Strong binding	Inhibition of receptor binding; 55% inhibition of the viral infection	[110]
	[HA-(101-257) from H9N2]		measured by ELISA		
				at 1nmol in the cell viability assay	
Influenza A virus	Hemagglutinin	HAS15-5; RNA	Strong binding	Inhibition of receptor binding	[111]
	(HA from H5N1)		using RT-PCR		
Influenza A virus	Hemagglutinin	D-26, 2' Fluoro modified RNA	67 fM	Inhibition of receptor binding, Complete inhibition of the agglutination of RBC in the presence of 200 nM aptamer	
	(HA from H1N1)				
Influenza B virus	Hemagglutinin (HA from B/Johannesburg strain)	A20; RNA	0.7 nM	Inhibition of receptor binding; 93% inhibition of viral fusion efficiencies in the presence of 25 µM aptamers	[112]
Rabies virus	Rabies virus (CVS-11) infected BHK-21 cells	F34; DNA	28 nM	Inhibition of CVS-11 infectivity with a dose-dependent manner	[113]
Rabies virus	Rabies virus (CVS-11) infected BHK-21 cells	PEG-FO24; PEGylated DNA	N.A.	87.5% survival rate of mice inoculated with aptamers for 24 h prior to challenge with rabies virus	[114]
HPV	HPV16 E7	G5α3N.4; RNA	1.9 µM	N.A.	[115]
HPV	HPV transformed HeLa cells	Aptamer 20, DNA	1 nM	N.A.	[116]
HPV	HPV16 E7	A2; RNA	107 nM	Induction of apoptosis in HPV infected cancer cells	[117,118]
HSV-1	Glycoprotein D protein	Aptamer-1; 2' Fluoro modified RNA	170 nM	Inhibition of gD protein and HSV-1	[119]
				target cell receptor (HVEM) interactions, EC_50_ = 60 nM	
HSV-2	Glycoprotein D protein	G7a; 2' Fluoro modified RNA	N.A.	Inhibition of receptor binding, IC_50_ = 20 nM	[120]

In 1992, Tuerk and Gold identified RNA aptamers against HIV-1 RT from an RNA pool that spanned a 32-nt random region by using *in vitro* SELEX [42]. The selected anti-RT aptamer contained a consensus sequence that resulted in the formation of an RNA pseudoknot. This RNA pseudoknot was reported to bind to HIV-1 RT in the picomolar range [63] and had an inhibitory effect on HIV-1 replication [64,121,122]. Since this discovery, numerous RNA or DNA aptamers of various lengths have been raised against HIV-1 RT and their uses for inhibiting the virus replication have been explored. For example, single stranded DNA aptamers (ODN112) with high affinity for the RNase H domain of HIV-1 RT were isolated by using recombinant RTs [66]. The selected DNA ligands could greatly diminish the infectivity of HIV-1 in human cells [66]. Similarly, double-stranded DNA thioaptamers (R12-2) that contained thiophosphate backbones were selected against the RNase H domain of HIV-1 RT by using *in vitro* combinatorial selection methods [67]. One lead thioaptamer could specifically bind to HIV-1 RT (dissociation constant (K_D_) of 70 nM) and significantly inhibited HIV-1 infection in a dose-dependent manner [67]. In addition to aptamers against wild-type (WT) HIV-1 RT, several RNA aptamers targeting a drug-resistant HIV-1 RT, mutant 3 (M3) have been isolated [65]. One of these aptamers, M302, bound M3, but did not have significant affinity for WT HIV-1 RT, which would likely allow specific detection of HIV-1 RT variants [65].

Anti-RT aptamers have been intracellularly expressed together with flanking, self-cleaving ribozymes to generate aptamer RNA transcripts that have minimal flanking sequences [122]. The expression of aptamers in the HIV-1 infected cell led to encapsidation of the aptamer in the virion particles, which subsequently blocked the HIV-1 replication [122]. These aptamers also effectively suppressed drug-resistant variants and other HIV subtypes (e.g., subtypes A, B, D, E, and F) [122]. More recently, Lange *et al*. further optimized this expression cassette, in which an extended, tertiary-stabilized hammerhead ribozyme was replaced to enhance its self-cleavage activity [123]. Stable clonal cell lines that expressed aptamers from these optimized constructs strongly suppressed infectious virus when used at a high multiplicity of infection (MOI) [123].

#### 2.1.2. HIV-1 Integrase

HIV-1 integrase (IN), a retrovirus encoded enzyme, catalyzes the insertion of proviral DNA into the host-cell genome [124]. Because HIV-1 IN is essential for retrovirus replication, it is a promising target for the development of antiretroviral drugs.

Soultrait *et al*. isolated several DNA aptamers (ODN93 and ODN112) that could strongly inhibit the RNase H activity associated with HIV-1 RT [74]. Because X-ray structure analysis indicated strong similarities between the RNase H domain of HIV-1 RT and the catalytic core of HIV-1 IN, the authors investigated if the truncated version of DNA aptamers against HIV-1 RT RNase H (ODN93del and ODN112del) could also bind to HIV-1 IN [125] and found that that was the case. The DNA anti-RT aptamers efficiently blocked the processing and strand transfer activity of HIV-1 IN when used at nanomolar concentrations *in vitro* and abolished HIV-1 replication in infected human cells [75]. Structural studies revealed the DNA aptamers contained G-rich sequences that could form stable and non-canonical G-quadruplex structure. A docking-based model of the OND93del-IN complex suggested that the DNA aptamer was located within a channel of the tetrameric integrase [126]. Because this mutual fitting could block several catalytic amino acid residues that are essential for integrase function, the binding site of the OND93del aptamer may contribute to its anti-HIV-1 activity. Additionally, various G-quadruplex aptamers (e.g., T30695 and AID-1) have proven effective for inhibiting HIV-1 IN *in vitro*, and are therefore regarded as potential anti-HIV therapeutic agents [127,128,129].

#### 2.1.3. HIV-1 Gag Protein

Gag polyprotein is a major HIV-1 structural protein that is synthesized in the cytoplasm of infected cells, orchestrates the assembly and release of HIV-1 particles, and is necessary and sufficient for the formation of noninfectious virus-like particles [130,131]. It is involved in both assembly and virion maturation after particle release, as well as early post-entry steps in virus replication. During virion maturation, Gag is cleaved into several component proteins: matrix protein (MA), capsid protein (CA), nucleocapsid protein (NC), the late domain (p6), and two small, spacer peptides, SP1 and SP2 [132]. These proteins play specific roles in the life cycle of HIV-1, and mutations in any domain of Gag lead to defects in particle assembly and loss of infectivity.

The abilities of RNA aptamers specific to the Gag polyprotein to inhibit HIV-1 replication have been explored [77]. Ramalingam *et al*. selected RNA aptamers against an HIV-1 Gag protein, lacking the N-terminal myristate and the C-terminal p6 (DP-6 Gag). The selected aptamers (DP6-22) showed strong binding affinity to the MA, NC or entire Gag protein. Binding studies revealed that the “NC-binder” could specifically compete with the packaging signal (ψ) of HIV-1 for binding to DP6-Gag [77]. Moreover, NC-binding aptamers disrupted Gag-genomic RNA interaction and negatively affected genomic RNA transcription, processing, or stability. The NC protein of HIV-1 plays an important role in encapsidating of viral RNA and assembling viral particles [133]. The NC protein specifically recognizes the viral genomic RNA through specific binding to the viral psi (ψ) sequences, and thus selectively encapsidates the viral RNA among different cellular RNA pools [134]. The NC is highly conserved and therefore is an excellent target for anti-viral therapy.

Kim *et al*. described the selection and stabilization of RNA aptamers against HIV-1 NC [78]. Surface plasmon resonance (SPR) analysis and gel shift assays showed that their selected RNA aptamer specifically bound to target NC protein with high affinity and competed with psi RNA binding to the NC protein [78]. Furthermore, the linear aptamer was tailored to form circular RNA, after which it showed higher stability and the same binding affinity to NC protein, thus providing a valuable inhibitor for viral packaging. In a similar study, an anti-NC RNA aptamer (N70-13) was shown to completely abolish NC binding to the stable trans-activation response hairpin and psi RNA stem-loops of HIV-1 RNA. When this aptamer was expressed in cells, it suppressed the packaging of viral genomic RNA [79,135].

#### 2.1.4. HIV-1 gp120 Protein

The envelope glycoprotein of HIV consists of an exterior glycoprotein (gp120) and a transmembrane domain (gp41) and plays an important role in viral entry into cells [136]. HIV-1 infection is initiated by interactions between HIV-1 gp120 and the human cell surface receptor CD4, subsequently leading to fusion of the viral membrane with the target cell membrane [137,138]. This interaction suggests HIV-1 gp120 as another potential therapeutic target to block HIV-1 infection.

RNA aptamers that bind the HIV-1 gp120 protein have been shown to neutralize HIV-1. For example, using SPR, James and colleagues developed several 2'-F-modified anti-HIV gp120 RNA aptamers that block the interaction of gp120 and CD4 [80,139]. Moreover, mutation analysis and functional studies of these aptamers demonstrated that they specifically interact with the conserved coreceptor region of an R5 stain derived gp120 and neutralize HIV-1 infectivity in human peripheral blood mononuclear cells [140,141]. The selected aptamers suppressed the infectivity of R5 clinical isolates of HIV-1 derived from group M (subtypes A, C, D, E and F) and group O. In addition, the researchers conducted a detailed characterization of one neutralizing aptamer, B40 (117-nt). They found the minimal region of the aptamer essential for binding gp120 comprises a three-way junction linking two helix-loops and a closing helix [141]. Accordingly, a 77-nt truncated version of the aptamer (B40t77) was designed, which efficiently prevented gp120 from interacting with host cells [142]. Recently, several shortened synthetic derivatives of the B40 aptamer were designed and assessed for their anti-viral activity. One such aptamer, UCLA1, tightly bound to a consensus HIV-1 subtype C gp120 and neutralized isolates of the same subtype with 50% inhibitory concentrations (IC_50_) in the nanomolar range and without causing toxicity in tested primary cells [81]. Furthermore, when UCLA1 was combined with T20, a gp41 fusion inhibitor, and IgG1b12, an anti-CD4 binding site monoclonal antibody, a synergistic effect was observed, suggesting UCLA1 may be a potential adjuvant anti-HIV agent [81].

**Figure 2:**
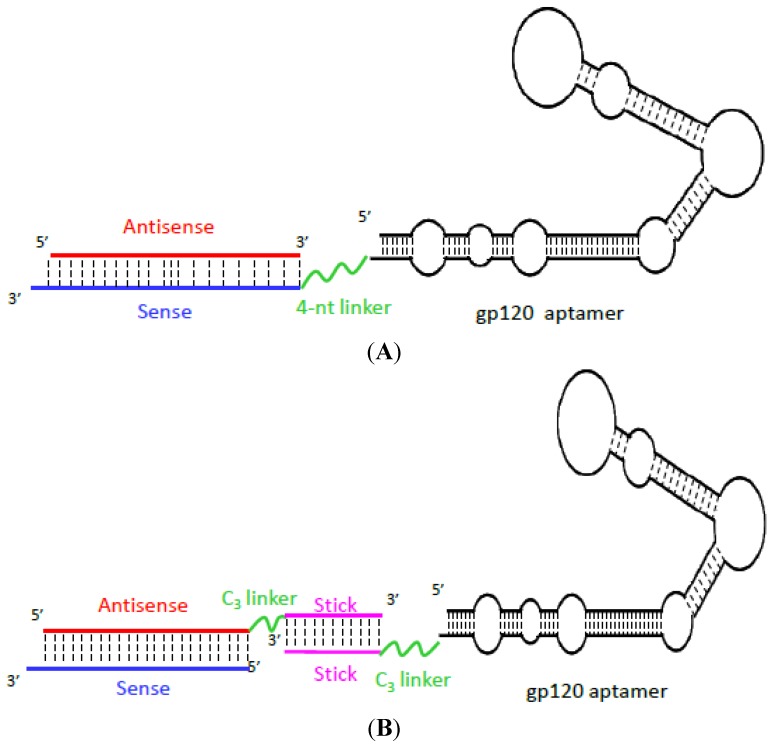
(**A**) Aptamer-siRNA conjugates. The 2'-F-modified gp120 aptamer was covalently appended to the sense strand of a *tat/rev* siRNA portion, which in turn was hybridized to the antisense strand. A 4-nt linker (CUCU) was inserted between the aptamer and siRNA portions to minimize steric interference of the gp120 aptamer with Dicer processing [143]. (**B**) Aptamer-stick-siRNA chimeras. The 2'-F-modified gp120 aptamer and the siRNAs are shown. The antisense of the siRNA is linked to the aptamer portion by the stick sequence, which consists of 16 nt appended to the 3' end of the gp120 aptamer, allowing complementary base-pairing of one of the two siRNA strands with the aptamers [83].

Recently, using a nitrocellulose membrane based *in vitro* SELEX procedure on an RNA library, our group also succeeded in selecting several 2'-F substituted RNA aptamers that bind to HIV-1_Bal_ gp120 protein with nanomolar affinity [83]. The evolved anti-HIV-1 gp120 aptamers efficiently inhibited the replication of CXCR4-tropic (NL4-3 and IIIB) and CCR5-tropic (BaL and JRFL) HIV-1, as well as several clinical HIV-1 isolates [50], and were specifically internalized by cells that expressed HIV-1 gp120. By taking advantage of the gp120 binding properties of an anti-gp120 aptamer, A-1, we tested the concept of using the gp120 aptamer to deliver anti-HIV-1 small interfering (si)RNAs into HIV-1 infected cells, with the goals to enhance therapeutic efficacy and reduce the off-target effect of siRNAs. siRNA is being exploited as a new class of medicine for a variety of diseases to inhibit expression of complementary RNA transcripts, but its clinical translation is dampened by specific cell delivery and internalization. In the chimera approach, we made two designs of gp120 aptamer-Dicer substrate siRNA (DsiRNA) conjugates for cell type-specific siRNA delivery [83]: A covalent aptamer-siRNA chimera and a non-covalent aptamer-stick-siRNA chimera (Figure 2). In the covalent aptamer-siRNA chimera (Figure 2A), we first identified that the anti-gp120 aptamer provided HIV-1 neutralizing activity, even with the siRNA conjugation, and served as a vehicle for selectively delivering anti-HIV-1 siRNAs into HIV-1 infected cells [50]. The activities of the anti-gp120 aptamer-siRNA chimera were further evaluated in a humanized RAG-hu mouse model of HIV-1 infection. The chimera significantly reduced the plasma viral load and maintained CD4 levels equivalent to those of uninfected control mice [143]. In the case of the aptamer-stick-siRNA chimera (Figure 2B), a single gp120 aptamer was used in combination with three different siRNAs that targeted HIV-1 *tat*/*rev* transcripts and HIV-1 host dependency factors (CD4 and TNPO3), resulting in knockdown of target mRNAs and potent inhibition of HIV-1 replication, as well as protection against viral-induced CD4+ T-cell depletion *in vivo* [83,144]. The advantage of the stick design facilitated the effective interchange of different siRNAs with a single aptamer, which is required to avert viral resistance to the siRNA component [24]. A follow-up treatment with the aptamer-cocktail DsiRNA conjugates after viral rebound resulted in complete, long-term suppression of HIV-1 viral loads, suggesting a facile, targeted approach for combinatorial delivery of antiviral and host siRNAs for HIV-1 therapy *in vivo* [50,144].

#### 2.1.5. HIV-1 Tat Protein

The HIV-1 viral trans-activator Tat protein consists of 86 amino acids, binds to specific regulatory elements (trans activation responsive [TAR] elements) in the HIV-1 long-terminal repeats (LTRs) and regulates viral transcription [145,146]. Tat also influences the growth and metabolism of host cells, and is important for the efficient reverse transcription of HIV-1 [147]. A 37-mer RNA aptamer, RNA^Tat^ that bound efficiently to the Tat protein of HIV-1, was isolated through *in vitro* selection procedure [84]. Compared to authentic TAR RNA, the dissociation constant of RNA^Tat^ to Tat was 133-times higher [84]. Because the RNA^Tat^ aptamers showed high affinity to Tat and did not interact with cellular proteins, it is likely to be a more efficient and suitable decoy than cellular TAR RNA, which interacts with several cellular factors within cells and inhibits the transcription of various genes [84]. The results demonstrated that the RNA^Tat^ aptamers could specifically prevent Tat-dependent trans-activation both *in vitro* and *in vivo*.

#### 2.1.6. HIV-1 Rev Protein

HIV-1 Rev, a virally encoded sequence-specific RNA-binding protein, plays a critical role in the nuclear export of intron-containing HIV-1 RNA. Rev shuttles between the nucleus and the cytoplasm, and harbors both a nuclear localization signal and a nuclear export signal [148,149]. Rev directly binds to unspliced and incompletely spliced viral RNA through the *cis*-acting Rev response element sequence [150].

RNA aptamers have been raised against the HIV-1 Rev protein through *in vitro* evolution [18]. Structural analysis and chemical modifications of these aptamers revealed that two major regions of the RNA aptamers were responsible for interacting with the Rev protein. These regions might provide a starting point for developing therapeutic nucleic acids for HIV-1 therapy [85]. Furthermore, the Rev-binding aptamer and the anti-Env ribozyme were constructed with the RSV and CMV promoters, which have a high transcription rate, produce a stable transcript and localize the transcript within the cytoplasmic compartment [151]. It is expected that the resulting cassette could effectively suppress HIV-1 production from HIV proviral clones.

#### 2.1.7. HIV-1 TAR Element

The HIV-1 TAR element, a 59-nt-long stem loop present at the 5' end of HIV-1 RNA, mediates the trans-activation of transcription through binding of the viral protein Tat [152]. The interaction between TAR RNA and Tat protein increases the processivity of the RNA polymerase, allowing high yield synthesis of the full-length retroviral genome [149,153]. Therefore, the binding of an oligonucleotide ligand (such as an aptamer) could be able to compete with the interaction between TAR RNA and Tat, which could prevent transcription.

Boiziau *et al*. performed *in vitro* selection to identify ligands specific to HIV-1 TAR [86]. After 15 rounds of selection-amplification, several DNA aptamers were isolated. Structural analysis revealed that the selected DNA aptamers (IV04) could fold into imperfect stem-loop structures and presented a consensus motif (5'-ACTCCCAT-3') [86]. The six central bases of the consensus were complementary to the TAR apical region, allowing the formation of RNA-DNA kissing complexes in the presence of 10 mM magnesium [86]. These complexes did not interfere with TAR function and were not observed when the magnesium concentration was lowered to a more physiological concentration (3 mM). It was supposed that the aptamer that had a lower binding affinity could not efficiently compete with the viral or cellular proteins that induce transcription. Therefore, the researchers added a counter-selection step to eliminate those binders that had lower affinity [89]. One of the aptamers could be further shortened to a 19-mer DNA and had a dissociation constant of 50 nM, but their inhibitory capabilities were not significantly altered [89].

Similarly, RNA aptamers against HIV-1 TAR have also been developed [87]. The selected sequences (R06_24_) folded into imperfect hairpins and displayed a consensus motif (5'-GUCCCAGA-3') that was complementary to the entire TAR loop, leading to the formation of TAR RNA-aptamer “kissing complexes” [87]. The lower nanomolar dissociation constant (K_D_ = 30 nM at 3 mM magnesium) of the selected RNA aptamers was attributed to closing of the aptamer loop by the consensus G and A residues [87]. Moreover, anti-TAR RNA aptamers were isolated from a genomic human library [88]. Theoretically, a genomic SELEX library contains all cellular RNA transcripts that can identify putative functional interactions involving RNA motifs [154]. The genomic SELEX is useful when studying for RNA-RNA interaction and searching for RNAs that might be expressed in a very low level. In the case of TAR aptamers, one of the selected aptamers, (a1), formed a complex with TAR through a kissing interaction and had ~4-fold lower dissociation constant than that of a previously identified aptamer, R06 (K_D_: 4 nM *vs*. 17 nM) [88].

Virus-resistant transgenic T cells and macrophages that express HIV-1 TAR aptamer either alone or in combination with others nucleic acid therapeutics have been produced by lentiviral gene transduction of CD34^+^ progenitor cells. When the differentiated T lymphocytes and macrophages were challenged with HIV-1, marked resistances against HIV-1 infection was seen [58]. For example, a triple combination lentiviral construct composed of a U6-driven TAR RNA decoy, a U6-promoted HIV-1 *tat/rev* shRNA, and a VA1-promoted anti-CCR5 trans-cleaving hammerhead ribozyme efficiently transduced human progenitor CD34^+^ cells [155]. These transduced cells had increased suppression of HIV-1 over 42 days when compared to cells that received a single anti-*tat/rev* shRNA or double combinations of shRNA/ribozyme or decoy [155]. Recently, this triple construct has been used for *ex vivo* gene delivery to hematopoietic stem cells in a human clinical trial [156]. The lentivirally transduced cells successfully engrafted in all four infused patients by day 11 and no unexpected infusion-related toxicities were seen [156]. Long-term (up to 24 months) expression of an ectopically expressed shRNA and ribozyme in multiple peripheral blood cell lineages of two of the transplanted patients was observed [156].

### 2.2. Inhibition of Hepatitis C Virus

Hepatitis C infection is a serious public health problem that chronically affects 170 million people worldwide [157]. Infection with hepatitis C virus (HCV) places individuals at high risk for scarring of the liver and ultimately leads to cirrhosis and liver cancer. HCV has limited therapeutic options, including a combination therapy of PEGylated interferon-α and ribavirin, which is not effective against certain HCV genotypes [158]. Thus, there is a need for new HCV therapies. The HCV is a 9.6 kb enveloped virus with a positive linear, single stranded sense RNA genome that contains a 5' non-translated region (NTR), protein coding region and 3' NTR for viral replication [159]. The 5' coding region consists of a single polyprotein of 3,010 amino acids, which is further processed by viral and cellular proteases into structural (C, E1, E2 and p7) and nonstructural (NS2, NS3, NS4A, NS4B, NS5A and NS5B) proteins [159]. Because some of these proteins are considered important for viral replication and proliferation, they are prime targets for the development of antiviral therapies.

#### 2.2.1. HCV Non-Structural Protein (NS) 3

The NS3 protein of HCV is a multi-functional enzyme that has two distinct domains: an amino (*N*)-terminal with trypsin-like serine protease activity that comprises one-third of the protein, and a carboxyl (*C*)-terminal with an NTP-dependent RNA/DNA helicase unwinding activity in the remaining two thirds. Generally, the protease activity of the NS3 cleaves the junctions between the non-structural proteins [160], while the helicase activity unwinds the double-stranded RNA generated by the RNA-dependent RNA polymerase NS5B during genome replication [161]. Because NS3 is essential for the HCV life cycle, many aptamers have been evolved against NS3 as potential anti-HCV agents. Kumar *et al*. isolated RNA aptamers from a randomized pool comprising 120 bases [90]. The selected aptamers, G6-16, bound specifically to the protease domain of NS3 and inhibited its activity *in vitro* [90]. Evaluation of enzyme kinetics revealed that the G6-16 aptamers inhibited the protease activity in a mixed (competitive and non-competitive) fashion with respect to the substrate [90]. Interestingly, the aptamers moderately inhibited the helicase activity of NS3, even though the aptamer bound to the protease domain of NS3 [90]. Although these aptamers have potential to serve as a dual-function inhibitor, their inhibitory concentrations were high (K_i_ = 3 µM) and appear to be significantly higher than those of other aptamers that target other proteins.

Because the G6-16 aptamers had low efficacy, a new set of aptamers against the NS3 proteins was re-evolved, using a slightly modified selection strategy [91]. A truncated protease domain of NS3 was used as a selection target instead of the entire NS3 protein, and a 30-nucleotide randomized core region was used for SELEX instead of a 120-nucleotide randomized core region [91]. Consequently, three classes of conserved aptamers were identified after 9 rounds of selection. The dissociation constant of these aptamers was about 10 nM, which could inhibit 90% of the protease activity of both truncated NS3 and entire NS3 in a non-competitive manner [91]. Mutational analysis of the aptamer G9-1 revealed that the sequence required for protease inhibition was in stem I, stem III and loop III of the aptamers [91]. Use of these aptamers *in vivo* required that they may be expressed intracellularly. Therefore, an *in vivo* aptamer expression system was designed that combined the G9-1 aptamer with *cis*-acting genomic human hepatitis delta virus (HDV) ribozymes to construct a chimeric HDV-ribozyme-G9-aptamer [162]. The G9-1 aptamer was inserted into the non-functional stem IV region of the HDV ribozyme so that the aptamer would be protected from exonuclease degradation in the presence of the tightly packed structure of the HDV ribozyme and hence remains stable and functional in cells [162]. Furthermore, the chimera was attached with a nuclear export signal (CTE-M45) and arrayed as a tandem repeat in the expression plasmid in order to increase its cytoplasmic level in cells. Evaluation of this expression system in cultured cells showed efficient protease inhibition activities similar to the aptamer G9-1 alone [162].

The HCV NS3 helicase has been reported to preferentially bind to poly U sequence. Therefore, Fukuda *et al*. added a poly U tail to the 3' end of the G9 aptamer [163]. Addition of the poly U tail not only increased binding and protease inhibition, but also resulted in an additional ability to inhibit the helicase. As compared with the original G9 aptamer, the modified G9-poly U aptamer had more potent protease activities and efficiently inhibited unwinding of DNA by the NS3 helicase [163]. The poly U tail is similar to a decoy that masks the substrate binding site of the NS3 helicase. Unfortunately, the poly U tail had only a moderate IC_50_ (1 µM) for inhibiting the NS3 helicase. Therefore, another SELEX against the HCV NS3 helicase domain was performed to identify a specific HCV NS3 helicase aptamer with and improved IC_50_ [92]. After 8 rounds of selection, aptamer 5 most strongly inhibited helicase activity *in vitro* (IC_50_ of 50 nM) [92]. Although HCV NS3 helicase uses NTPs as an energy source to unwind nucleic acids, only the helicase activity of the protein was inhibited by the aptamer 5, while the NTPase activity was not affected, suggesting that the aptamer may bind to the nucleic acid binding site of the helicase to block the unwinding activity [92]. Furthermore, given the identification of specific and effective aptamers against the protease and helicase domains of HCV NS3, a dual-function nanoparticle was created by conjugating the protease aptamer G9 and the helicase aptamer 5 through a poly U spacer [164]. The distance between these two aptamers was carefully adjusted. The resulting chimera, G925-s50, was more effective at inhibiting NS3 protease/helicase activity *in vitro*. In particular, the helicase activity had four-fold greater inhibition when compared with aptamer 5 alone [164]. Cell culture experiments further demonstrated that the G925-s50 chimera effectively inhibited NS3 protease activity in living cells and in an HCV genome replication system [164].

#### 2.2.2. HCV Non-Structural Protein 5B

The NS5B viral polymerase of HCV is an RNA-dependent RNA polymerase that is essential for transcribing the full length of the HCV genome [165]. During synthesis of viral RNA, NS5B synthesizes a negative strand RNA that serves as a template for the synthesis of new positive RNA strands. This essential role of NS5B in the HCV life cycle has made it attractive for the development of anti-HCV agents. Biroccio *et al*. isolated an RNA aptamer against a truncated NS5B protein that lacked the C-terminal membrane localization signal (NS5B-∆C55) [93]. Deletion of this C-terminal sequence helped to enhance stability and catalytic activity of the protein to improve the SELEX procedure. The selected aptamers shared a consensus sequence that can fold into a stem-loop structure [93]. In particular, one aptamer, B.2, bound to the NS5B protein with high affinity and non-competitively inhibited the activity of NS5B polymerase with respect to template RNA [93]. This observation suggested that the B.2 aptamers and template RNA did not bind to the same region of the protein. Similarly, Bellecave *et al*. identified two DNA aptamers (27v and 127v) that specifically bound to NS5B and inhibited its polymerase activity [94]. However, these aptamers used two different mechanisms to inhibit the enzyme. The 27v aptamer competed with template RNA and inhibited both steps of RNA synthesis (*i.e.*, initiation and elongation), while the 127v aptamer competed poorly with the RNA template and inhibited only initiation [94]. Further cell culture studies revealed that the 27v aptamer could inhibit viral RNA synthesis and interfered with viral particle production in HCV JFH1 infected Huh7 cells [94].

Other HCV NS5B aptamers include 2' F modified aptamers evolved by Lee *et al*. Transfection of this aptamer into cells significantly suppressed replication of both HCV genotype 1b and 2a, but did not generate escape mutant viruses or cause cellular toxicity [95]. This potent aptamer was further modified through conjugation of cholesterol or galactose-polyethylene glycol ligands to increase its stability and specificity for the liver.

#### 2.2.3. HCV Internal Ribosome Entry Site

**Figure 3:**
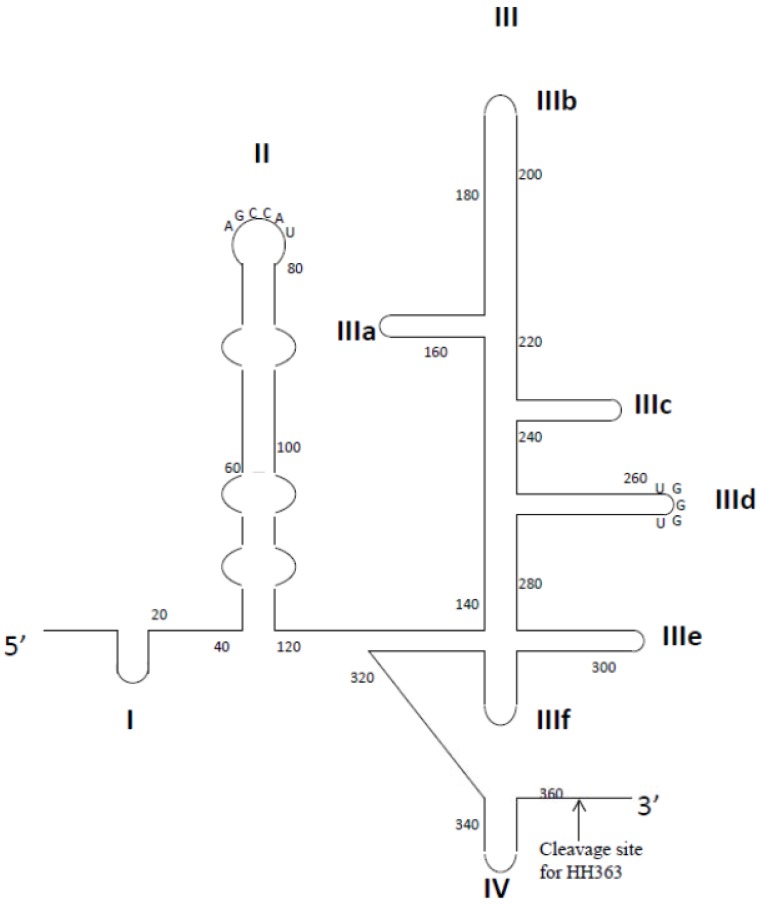
Representation of the secondary structure of HCV IRES. Structural domains are shown as I–IV. The HH363 cleavage site is indicated by an arrow.

The internal ribosome entry site (IRES) is a highly conserved and complex RNA structure located at the 5' non-translated region of the HCV genome. The HCV IRES is associated with the host cell small ribosomal unit (40S) and eukaryotic initiation factor 3 (eIF3) to mediate translation initiation of the HCV genome in a 5' cap independent manner [166]. The IRES RNA has four conserved domains (I-IV) for which the structure-function relationships are well-understood (Figure 3) [167]. To identify an RNA aptamer against domain II of IRES, Kikuchi *et al*. used a biotinylated DNA probe that was complementary to the 5' extension of domain II to fix the IRES on streptavidin magnetic beads [97]. After 4 rounds of selection, almost all clones contained the conserved sequence 5'-UAUGGCU-3', which is complementary to the apical loop of domain II [97]. This observation suggested that the apical loop of domain II may be exposed to interact with cellular molecules such that it is accessible to contact with aptamers. One aptamer, 2-02, had a low dissociation constant to IRES domain II (K_D_ of 11 nM), but this aptamer only moderately inhibited the IRES-dependent translation [97]. Similarly, the research group isolated RNA aptamers capable of binding to domain III-IV [98]. These aptamers also shared the conserved sequence 5'-ACCCA-3', which is complementary to the apical loop of domain IIId [98]. This loop is known to be a critical region for IRES-dependent translation. Notably, the 3-07 aptamer showed very high affinity to domain III and strongly inhibited the IRES-dependent translation of firefly-luciferase mRNA in mammalian cells [98]. Furthermore, conjugates of domain II-specific 2-02 aptamers and domain III-specific 3-07 aptamers not only bound to the IRES more efficiently than the two parental aptamers, but also inhibited IRES-dependent translation about 10 times as efficiently as the 3-07 aptamer [168].

Romero-Lopez *et al*. developed an innovative aptamer-ribozyme chimera that comprised an aptamer targeted to domain IV of the IRES and a hammerhead ribozyme that targeted the HCV genome [99]. To identify a chimera that contained both cleaving and binding activity, an RNA library pool was first designed on the basis of the well-characterized hammerhead ribozyme that cleaved at the position 363 of the HCV genome [99]. A 25-nt long randomized region was attached at the 3' end of the ribozyme. Identification of potent chimera then involved two steps: selection of aptamers that specifically bound to the IRES and identification of high-affinity aptamers that retained catalytic activity for cleaving HCV RNA [99]. Consequently, a chimeric molecule (HH363-50) composed of two inhibitors was identified. HH363-50 actively functioned as a chimeric inhibitor *in vitro*, suggesting that conjugation of the aptamer and ribozyme domains did not cause any loss of function [100]. The aptamer domain bound to domain IV of IRES and interfered with formation of a productive 80S ribosomal complex, while the ribozyme domain reduced the HCV RNA levels in a subgenomic replicon system by cleaving the viral genome [100]. These studies confirmed the success of combining different functional nucleic acids to produce dual-function inhibitors.

### 2.3. Inhibition of Hepatitis B Virus

Infection with hepatitis B virus (HBV) is one of the most prevalent chronic infections associated with serious clinical outcomes, including hepatitis, cirrhosis and liver cancer. It is estimated that HBV infection affects approximately 350 million people worldwide, but available therapies against HBV infection are limited in term of their efficacy and safety. Licensed interferon-α and nucleoside analogs, such as adefovir and lamivudine, are routinely used to treat chronically infected patients. However, although these drugs have therapeutic effects, their use is limited by the development of resistant HBV strains and unwanted side effects [169,170].

The HBV genome contains an unusual partly double stranded relaxed circular DNA (rcDNA), meaning that the (−) strand DNA encompasses the entire HBV genome, while the (+) strand DNA is shorter and variable in length [171]. During disease progression, rcDNA forms a covalently closed circular DNA in the nucleus to allow transcription of HBV RNAs, which are capped and polyadenylated [171]. The viral genome encodes four open reading frames, core proteins (C), viral reverse DNA polymerase (P), surface antigen (S) and X protein (HBx) [171]. Several proteins encoded by HBV have been targeted for aptamer selection.

#### 2.3.1. HBV Surface Antigen

HBV surface antigen (HBsAg) is situated on the membrane of HBV. During HBV replication, HBsAg is overexpressed on the surface of HBsAg^+^ hepatocytes. Because of this, HBsAg is often used as a target for HBV diagnosis and therapy. Liu *et al*. selected RNA aptamers against purified HBsAg protein from an initial 115 mer RNA library of 10^15^ sequences [101]. The HBs-A22 aptamers bound specifically to the HBsAg^+^ hepatoma cell line HepG2.2.15, but not to HBsAg-devoid HepG2 cells [101]. Although the aptamer was shown to bind to a HBV surface antigen, their binding affinities, potential cross-reactivity with the M and L versions of the surface antigen and their abilities to interfere with particle assembly were not described.

#### 2.3.2. HBV Polymerase

The specific interactions between HBV polymerase (P) protein and the ε RNA stem-loop at the 5' end of the HBV pregenomic (pg) RNA are a unique characteristic of HBV replication [172,173]. These interactions are required for the assembly of replication-competent capsids. Beyond HBV replication, formation of the P-ε complex is necessary for protein-primed reverse transcription of an RNA intermediate [173,174]. Thus, molecules that inhibit these interactions should block viral replication at both the pgRNA packaging and reverse transcription levels. Recently, Feng *et al*. used SELEX to identify potential ε decoys in a RNA library; they sought decoys that mimicked the secondary structure of the natural ε RNA [102]. The ε RNA contains a lower stem, an upper stem, a bulge and an apical loop [175]. The screening library was designed such that the 23-nt upper stem of the RNA pool was randomized, while sequences for the lower stem, the bulge and the apical loop were preserved. After three rounds of selection against truncated P protein, the S9 aptamer displayed higher binding to truncated P protein than did wild-type ε RNA [102]. Also, cotransfection of the S9 RNA vector with an HBV expression plasmid reduced the amount of viral replicative DNA intermediates by 80%–85% [102]. The S9 aptamer strongly inhibited the pgRNA packaging and DNA synthesis steps of HBV replication, suggesting that it acts as an ε decoy that competes with natural ε pgRNA upon binding to the P protein [102]. Notably, there was no sign of cytotoxicity in S9 aptamer-transfected cells.

### 2.4. Inhibition of Severe Acute Respiratory Syndrome Coronavirus

Severe acute respiratory syndrome (SARS) is a life-threatening form of pneumonia that caused almost 800 deaths between 2002 and 2003 [176]. The causative agent of SARS is the SARS coronavirus (SCoV), which is an enveloped, single-stranded RNA virus with a genome of ~30 kb [176]. Within weeks of the start of the outbreak, the SCoV genome structure had been determined; it encodes two large replicative polyproteins, pp1a and pp1ab, and structural proteins including spike (S), membrane (M), envelope (E), and nucleocapsid (N) proteins [177]. The four structural proteins generate the virus particle. Although infection control measures and surveillance successfully contained the spread of SCoV in humans, there is still no effective therapeutic regimen against the virus.

The SCoV helicase (nsp13) is necessary for viral replication and proliferation, and thus offers an attractive target for development of anti-SCoV aptamers [178]. Two studies independently isolated potent nucleic acid aptamers, both DNA and RNA, against the protein. These aptamers demonstrated sub-nanomolar IC_50_ values against the helicase [103,104]. Interestingly, the SCoV helicase contains a functional domain with double-stranded nucleic acid unwinding and ATPase activities. However, only the nucleic acid unwinding activity of the protein was inhibited by aptamers, while the ATPase activity was not affected, suggesting that the aptamer may bind to the nucleic acid binding site of the helicase and block the unwinding activity [103,104]. Intriguingly, this phenomenon is observed in HBV and HCV helicase inhibition mediated by aptamers. Although both DNA and RNA aptamers against SCoV helicase were selected, no similarity was seen in their sequences. Recently, a newly discovered coronavirus, namely Middle East respiratory syndrome coronavirus (MERS-CoV), was identified in Saudi Arabia that produced clinical symptoms resembling SARS [179]. Both MERS-CoV and SCoV belong to the genus beta-coronavirus [180]; however, they are genetically distinct and their replicase domains only have less than 50% amino acid identity [181]. So, it is unlikely that the SCoV helicase aptamers will be used for the treatment of MERS-CoV infection.

### 2.5. Inhibition of Influenza Virus

Influenza is considered the most prevalent infectious disease in humans. Over the past century, this virus has accounted for three major pandemics, which cumulatively killed tens of millions of individuals [182]. In particular, H1N1 influenza, also known as Spanish flu, killed more than 20 million people around the globe [183]. Recently, the emergence of H7N9 and H5N1 in China has raised concerns [184,185]. Fortunately, no sustained person-to-person spread of these viruses has been confirmed to date.

There are three major genetically distinct influenza viruses (types A, B and C). Influenza A, which can infect various animal hosts, has a high evolutionary rate and has been responsible for many of the major flu pandemics. On the other hand, influenza B and C, which usually cause relatively mild illnesses, are of less concerns [182]. Generally, influenza A viruses are enveloped, single-stranded RNA viruses that contain eight negative strand RNA segments and two membrane glycoprotein components, hemagglutinin (HA) and neuraminidase (NA) [186]. Mutations (antigenic drift) and re-assortment of genome segments (antigenic shift) have led to the evolution of various strains of influenza. These strains are classified according to the H number (for HA) and the N number (for NA). For example, an “H5N1 virus” designates an influenza A virus subtype that has an HA 5 protein and an NA 1 protein. The HA and NA proteins have served as prime targets for several of FDA-approved small molecules and vaccines [187,188,189]; however, because influenza A is highly adaptive and these drugs are widely used, there are now strains that are resistant to these treatments.

The HA antigen is ubiquitously expressed (~900 copies per virus) on the surface of each viral particle and is required for binding and membrane fusion with host cells to mediate the early stage of viral infection [190]. So far, eight aptamers against influenza viruses have been developed for therapeutic purposes, and all of these aptamers target HA (Table 1). In an early study, Jeon *et al*. evolved DNA aptamers against a HA peptide (position: 91–261) that had a conserved receptor binding pocket among all H3 subtypes [105]. Based on a hemagglutination assay and enzyme-linked immunosorbent assay (ELISA), the A22 aptamer was found to be the most efficient at blocking hemagglutination of various H3N2 strains (A/Texas/1/77, A/Japan/57 and A/Port Chalmers/1/73) when used at picomolar ranges [105]. Moreover, the A22 aptamers not only inhibited the hemagglutinin capacity of the virus, but also prevented the viral binding and entry to cells [105]. Importantly, intranasal administration of the A22 aptamer into influenza-infected mice reduced the virus burden by 95%, which is comparable with the amount of inhibition seen in mice after treatment with NA-based drugs currently available in the clinic [105].

In a similar study, Gopinath *et al*. devised a novel selection strategy that used the whole virus, instead of purified proteins, as a selection target to isolate aptamers [107]. In this approach, an H3N2 subtype (A/Panama/2007/1999) was initially incubated with the RNA library, followed by counter-selection with another subtype of H3N2 A/Aichi, which is closely related to A/Panama/2007/1999 [107]. In this way, the aptamers (P-30-10-16) were identified as blocking viral-cell interactions by binding to the HA of the target strains and failing to recognize other influenza A subtypes [107]. Although there are many parallels between aptamer and antibody technology, this study clearly demonstrated that the isolated aptamers displayed different molecular recognition mechanisms and had >15-fold higher binding affinity to the HA in comparison with the commercially available anti-HA monoclonal antibody [107]. These aptamers not only have the potential to be developed as therapeutic agents, but could be used as genotyping reagents for identifying different influenza subtypes. Recently, Gopinath *et al*. isolated 2'-fluoro modified aptamers that specifically targeted the HA protein of H1N1 viruses (A/California/07/2009). One aptamer, D26, was further optimized and displayed strong binding affinities, as determined by surface plasmon resonance. Moreover, this aptamer could distinguish the HA of H1N1 from other subtypes and interfere with HA-glycan interactions.

Although the influenza A and B have different biological mechanisms, as well as evolutionary characteristics and genetic lineages, they are largely similar in the symptoms they cause during infection [191]. Therefore, aptamers that can specifically recognize influenza B virus would be very useful for both therapeutic and diagnostic perspectives. Similarly, RNA aptamers specific for influenza B were isolated against the influenza virus B/Johannesburg/05/1999. The isolated aptamers bound specifically to the HA of influenza B virus, but not to that of influenza A virus [112]. Interestingly, HA recognition by these aptamers required magnesium ions in order to inhibit HA-mediated membrane fusion [112].

### 2.6. Inhibition of Rabies Virus

Rabies is a viral disease that causes nerve damage and death in many mammals. The causative agent of rabies is the rabies virus (RABV), which can be transmitted between species, often through saliva transferred during a bite from a rabid animal. Currently, there is no effective treatment for rabies once symptoms develop, and death occurs within weeks. Although post-exposure rabies treatments are available to treat RABV-infected patents before the virus invades the central nervous system, the virus still causes about ~60,000 deaths annually worldwide [192].

Liang *et al*. twice used a cell-SELEX approach to attempt to isolate DNA aptamers against RABV. In the first study, aptamers were isolated through 35 iterative rounds of selection [113]. The library was first incubated with rabies virus (CVS-11)-infected baby hamster kidney (BHK)-21 cells, followed by counter-selection of uninfected BHK-21 cells. This process identified five aptamers that inhibited replication of RABV [113]. Although the aptamers bound very tightly to the virus, their inhibitory capacity was limited and they did not cross-react with other rabies strains. This led the group to use the same cell-SELEX approach to select a new batch of aptamers. In their second attempt, 16 DNA aptamers were isolated, but only the aptamer, FO21, inhibited significantly the replication of RABV but not other related viruses [114]. The FO21 aptamers were further modified with polyethylene glycol (PEG). *In vivo* testing showed that the aptamers effectively protected infected mice, enabling a 87.5% survival rate when mice were treated with aptamers for a day prior to challenge with RABV [114]. However, almost all mice died when they were challenged with RABV prior to treatment with aptamers because RABV replicates rapidly and produces a large amount of infectious particles once it enters the brain, making the aptamer ineffective [193].

### 2.7. Inhibition of Human Papillomavirus

Human papillomavirus (HPV) infects keratinocytes of the skin or mucous membranes, and is considered one of the most common sexually transmitted diseases. HPV causes 440 million infections and 288,000 deaths worldwide [194]. There are more than 180 genotypes of HPVs. Although the majority do not cause severe clinical outcomes, some types of HPV are linked to cervical and throat cancers [195,196]. For example, infection with HPV16 (50% of cases) or HPV45 (30% of cases) is strongly connected to the cause of cervical cancer [197,198], which is the second most common cancer in women, while HPV18 is commonly found in patients with head and neck cancer [199]. Recent progresses in HPV vaccines, such as Gardasil^®^ (Merck) and Cervarix^®^ (GSK, London, UK), are effective means to prevent infection with certain types of HPV, but are ineffective at treating those who are already infected [200]. In fact, because the HPV family is so large, it is challenging to design effective and universal prophylactic and therapeutic approaches against this disease [200].

All HPV nucleic acid aptamers reported to date targeted the E7 proteins for the therapeutic treatment of the high-risk HPV16 subtype. The HPV E7 gene is oncogenic and can cause malignant transformation of cervical cell lines, while downregulation of this gene induces programmed cell death (apoptosis) [201]. Hence, inhibiting this protein represents a rational way to kill HPV^+^ cervical carcinoma cells.

#### HPV16 E7

Several groups have selected nucleic acid to HPV16 E7 proteins and demonstrated similar results [115,117,118,202]. The roles of E7 in the cell are well-characterized. The E7 proteins interact with the cell cycle control protein pRb, inhibiting its binding with E2F transcription factors and, leading to unrestricted bypass of the G1-S check point of the cell cycle [201,203]. In addition, E7 has been shown to interact with a wide range of cellular factors, including TATA binding proteins and p300 [204,205]. Nicol *et al*. identified a functional RNA aptamer (A2) that disrupted the interaction between E7 and pRb [117]. Protein mutagenesis assay showed that this aptamer bound to the N-terminal residue of E7, which is known to be involved in the interaction with pRB [117]. Transfection of HPV-infected cancer cells with A2 aptamers resulted in loss of E7 and elevated pRb levels, leading to cell apoptosis [117].

### 2.8. Inhibition of Herpes Simplex Virus

Herpes simplex virus (HSV) often infects primary epithelial tissues before invading the nervous system, where it becomes latent. There are two forms of HSV, HSV1 and HSV2, which share 70% genome identity [206]. Both HSV-1 and HSV-2 are ubiquitous and highly contagious, but there is no effective treatment that can eradicate the virus totally from the body [207]. Akin to other antiviral drugs that can block viral entry, anti-HSV aptamers were evolved against a glycoprotein D (gd) of HSV-1 and HSV-2 [119]. The gD protein is an essential viral surface protein that is required for binding to the host cell receptors for viral entry and fusion [208]. In HSV-1 aptamer selection, an aptamer was evolved that can specifically bind to the HSV-1 gD protein using protein-based SELEX. Although the gD proteins of HSV-1 and HSV-2 are highly homologous (86% sequence identity) [209], the aptamer can discriminate between the two. Chemical modifications of the HSV-1 aptamers with 2' fluoro increased the stability of the RNA against nuclease degradation, but did not greatly affect their binding capacity [119]. Furthermore, the aptamer effectively inhibited HSV-1 entry to the cell by blocking the interaction between the gD protein and the HSV-1 target cell receptor [119]. Similarly, the HSV-2 aptamers were selected against the gD protein and counter-selected against IgG to remove non-specific binders [120]. A panel of aptamers was shown to neutralize HSV-2 infection dependent on two entry receptors, Nectin 1 and HVEM. One effective aptamer, G7a, inhibited HSV-2 infection in Vero cells with an IC_50_ of 20 nM and its inhibition required the ACCCA motif for binding and function [120]. Given the importance of HSV-2 infection in enhancing the risk of HIV-1 transmission, this aptamer is proposed to be incorporated into a microbicide to reduce the spread of HSV-1 as well as HIV-1.

## 3. Conclusions

Since the first publication of SELEX over two decades ago, the development of aptamer technology has advanced rapidly from the laboratory to early or mid-stage clinical development [210]. Aptamers, also described as chemical versions of antibodies, can inhibit their targets through specific and strong interactions that are superior to those of biologics and small molecule therapeutics, and yet avoid the toxicity and immunogenicity concerns of these traditional agents derived from their nucleic acid compositions [26]. The latest advances in SELEX technology and chemical conjugation methods have given aptamers remarkable potential to be used as “smart bombs” that delivers secondary therapeutic cargos to diseased cells. Several examples (e.g., aptamer-siRNA chimeras, aptamer-ribozyme chimeras and aptamer-aptamer chimeras) discussed in this review demonstrate complementary and versatile approaches for combining the strength of aptamers with other nucleic acid-based therapeutics, offering a polyvalent platform for treating various diseases [23,38,143]. These chimeras offer a huge potential to provide enhanced therapeutic potency and reduced cellular toxicity of the drug. However, despite the substantial advances described above, no aptamers have yet reached clinical development pipeline for antiviral therapy. Aptamers whose targets are expressed intracellularly are unlikely to be used in the clinic because aptamers are hydrophilic and therefore cannot pass through epithelia and the hydrophobic plasma membrane [211]. Consequently, only aptamers that target extracellular viral proteins or capsid proteins of virions, such as HIV-1 gp120 or influenza A HA, are likely to be suitable for clinical therapeutic development [211]. In addition to the aptamer chimera approach, another potential approach to solve this problem would be to use a viral vector that will transiently express the aptamer intracellularly. For example, Bai *et al*. designed lentiviral vectors that encode anti-HIV ribozymes together with anti-Tat aptamers [51]. The construct was tested in HIV-infected humanized mice and was able to inhibit virus replication [51]. However, it was not conclusive whether the aptamers contributed any inhibitory effect.

Furthermore, as typical nucleic acid entities, naked nucleic acid aptamers are relatively small and are sensitive to nuclease degradation. Their average diameter is usually less than 10 nm, and therefore they are rapidly removed from the blood by renal clearance [44]. Thus, the intrinsic physicochemical features of aptamers pose serious challenges for their transport to infected organs or cells, such as the liver and central nervous system, following systemic administration into the blood stream. Typically, respiratory viruses, such as influenza viruses and SCoV, are well-suited for targeting with aptamer therapeutics because the upper airways and lungs are relatively easy to access as target organs [1]. Therefore, it may be possible to block respiratory virus infections by using an aptamer-containing aerosol [211]. Similarly, sexually transmitted viruses, such as HIV-1 and HPV, might be targeted by intravaginal application of a microbicide or cream that contains the neutralizing aptamers [211]. Although a topical microbicide might protect women against the viruses before the intercourse, the female genital tract is abundant in various nucleases that can degrade nucleic acid aptamers, even the 2' F modified ones, in minutes [212]. One way to improve the aptamer stability is to chemically introduce the 2'-O-Me modifications on the purine nucleotides or phosphorothioate linkages [212]. Moreover, zinc ions can be incorporated into the formulation because nucleases are sensitive to inhibition by zinc ions [212]. Recently, Wheeler *et al*. developed a topical microbicide containing chemically modified CD4 aptamer-siRNA chimeras that target the HIV co-receptor CCR5, gag and vif for the protection from sexual transmission of HIV-1. The chimeras were stabilized and formulated in a hydroxyethyl cellulose gel, which is a FDA-approved polymer already used in HIV-1 clinical trials, to achieve durable gene knockdown and inhibit HIV-1 transmission in mice [213].

The use of aptamers as therapeutic agents is still in its early stage of development. However, the innovation and flexibility of SELEX methodology will allow aptamer technology to become a major player as an alternative approach in the battle against viral diseases.
